# Lack of association between polymorphisms of *MASP2 *and susceptibility to SARS coronavirus infection

**DOI:** 10.1186/1471-2334-9-51

**Published:** 2009-05-01

**Authors:** Yan Wang, Jiangwei Yan, Yuling Shi, Ping Li, Chuanxuan Liu, Qingjun Ma, Ruifu Yang, Xiaoyi Wang, Lina zhu, Xiao Yang, Cheng Cao

**Affiliations:** 1 Beijing Institute of Biotechnology, Beijing, PR China; 2 Affiliated Bayi Children's Hospital, General Hospital of Beijing Command of the People's Liberation Army, Beijing, PR China; 3 Beijing Institute of Genomics, Chinese Academy of Sciences, Beijing, PR China; 4 General Hospital of Guangzhou Command of the People's Liberation Army, Guangzhou, PR China; 5 Beijing Institute of Microbiology and Epidemiology, Beijing, PR China

## Abstract

**Background:**

The pathogenesis of severe acute respiratory disease syndrome (SARS) is not fully understood. One case-control study has reported an association between susceptibility to SARS and *mannan-binding lectin *(*MBL*) in China. As the downstream protein of *MBL*, variants of the *MBL*-associated serine protease-2 (*MASP2*) gene may be associated with SARS coronavirus (SARS-CoV) infection in the same population.

**Methods:**

Thirty individuals with SARS were chosen for analysis of *MASP2 *polymorphisms by means of PCR direct sequencing. Tag single nucleotide polymorphisms (tagSNPs) were chosen using pairwise tagging algorithms. The frequencies of four tag SNPs (rs12711521, rs2261695, rs2273346 and rs7548659) were ascertained in 376 SARS patients and 523 control subjects, using the Beckman SNPstream Ultra High Throughput genotyping platform.

**Results:**

There is no significant association between alleles or genotypes of the *MASP2 *tagSNP and susceptibility to SARS-CoV in both Beijing and Guangzhou populations. Diplotype (rs2273346 and rs12711521)were analyzed for association with susceptibility to SARS, no statistically significant evidence of association was observed. The Beijing and Guangzhou sample groups were homogeneous regarding demographic and genetic parameters, a joined analysis also showed no statistically significant evidence of association.

**Conclusion:**

Our data do not suggest a role for *MASP2 *polymorphisms in SARS susceptibility in northern and southern China.

## Background

Severe acute respiratory disease syndrome (SARS), a new and highly infectious disease that is caused by a previously undescribed coronavirus in humans, has created a major public health threat in many countries [1-3]. Progress has been made in understanding SARS coronavirus (SARS-CoV) and the epidemiology, clinical manifestations, laboratory findings and radiological features of this disease have all been studied in detail [4,5]. However, its pathogenesis is still not fully understood. It has been reported that diabetes mellitus and heart disease are risk factors for adverse prognosis of SARS [6], however, little is known about the contribution of genetic factors.

SARS has been found to have a profoundly adverse effect on the immune system [7]. Variation in host immunity may be one of the important factors that determine susceptibility to SARS. A few case-control studies have reported an association between SARS susceptibility and human leucocyte antigen (HLA) and *MBL *[8-11]. Deficiency of *MBL*, a key component of the innate immune system, has been detected in SARS patients. Such a deficiency may increase susceptibility to SARS infection. Three papers reported the association of HLA and susceptibility to SARS [8-10]. Of these, two reported the association of HLA with susceptibility and resistance to the development of SARS [8,9]. In a more recent paper, no statistical significance was found with the susceptibility and severity of the disease [10].

*MBL *is a member of the collectin family and plays an important role in innate immunity [12,13]. *MBL *and ficolins distinguish between self, non-self and altered-self by recognizing patterns of ligands on the surface of microorganisms or aberrant cells. When this happens, *MBL*-associated serine protease-2 (*MASP-2*) is activated and cleaves complement factors to initiate the antibody-independent pathway of the complement system, thus starting inflammatory reactions.

*MBL*-associated serine proteases interact with *MBL *via the collagenous region of larger *MBL *oligomers. Four related proteins derived from two genes have been reported; namely *MASP1*, its alternative splicing variant *MASP3*, and *MASP2 *with its alternative splicing variant *Map19 *[14-16]. *MASP2 *activates the complement system by cleaving complement proteins C4 and C2. *MASP2 *is an essential component of the lectin pathway of complement activation.

*MASP2 *deficiency is observed because of genetic polymorphisms. It is known that a *MASP2 *polymorphism, namely D120G has a functional effect on the protein, and does not allow the formation of an active *MBL*-*MASP *complex. This variant has been described for the first time in a patient with an inherited deficiency of *MASP2*, who was characterized by augmented susceptibility to infection and development of immunological disease.

With regard to SARS-CoV infection, the codon 54 variant of the *MBL *gene has been shown to be associated with infection susceptibility but not with disease severity [11]. As the downstream protein of *MBL*, variants of the *MASP2 *gene may be associated with SARS-CoV infection. To examine the hypothesis that polymorphisms of the *MASP2 *gene in SARS patients are genetic factors that influence infection susceptibility, we studied *MASP2 *gene polymorphisms in DNA from two groups of Chinese SARS patients, and compared these with normal blood donors from the same region.

## Methods

### Recruitment of subjects for case-control study

This study was performed with the approval of the Ethical Committee of the Chinese Human Genome Center. A total of 376 SARS patients, who were diagnosed based on WHO criteria, were recruited during the 2003 outbreak. All SARS patients selected for study were unrelated and were shown to be seropositive by anti-SARS-N protein ELISA and immunofluorecent assay (IFA). The specificity of the ELISA assay is more than 99% [17,18]. Of these, 272 were from the wards at Xiaotangshan Hospital, Beijing, China, and the remaining 104 were from the Eighth People's Hospital of Guangzhou in southern China. A total of 523 age, sex, and ethnicity-matched healthy, genetically unrelated and seronegative (confirmed by anti-SARS N protein antibody ELISA and SARS antibody IFA) adults were recruited as control subjects. We extracted genomic DNA from peripheral blood leukocytes of affected individuals and controls using the QIAamp DNA Mini Kit (Qiagen, Valencia, CA). DNA was quantified using standardized fluorometric reading on a DU 650 spectrophotometer (Beckman Coulter, Fullerton, CA). Each sample was diluted to a final concentration of 10 ng/ul.

### Discovery of polymorphisms

Genomic DNA from 30 individuals with SARS was chosen for analysis of *MASP2 *gene polymorphisms. The sample included 60 chromosomes, which provided a 95% confidence level to detect alleles with a frequency >5% [19]. With a candidate-gene strategy, we screened for polymorphisms in all exons (~2.0 kb), 5'- and 3'-flanking regions (lengths of 2.5 kb), untranslated regions (~0.3 kb), and about 2.5 kb intronic sequences. DNA sequence spanning the *MASP2 *gene was obtained from the National Center for Biotechnology Information (NCBI) website (available from http://www.ncbi.nlm.nih.gov/; NT_021937.18). Primers were designed using the Premier program **(**Primer Premier 5). PCR primer sequences see Additional file 1. DNA samples from the 30 individuals were amplified and purified. The PCR reaction volume was 50 μl, containing 10–50 ng of DNA,10 pmol of each forward and reverse primer, 0.2 mmol/L of each dNTP,0.3 U of DNA polymerase(Tiangen Biotec Co, Beijing, China),10 mmol/L of Tris-HCl, 1.5 mmol/L of MgCl_2_, 50 mmol/L of KCl, and 0.1% Triton X-100. Amplication was carried out in a GeneAmp PCR system 9700(ABI) with cycle parameters of 5 min 94°C (initial denaturation), 32 rounds of 94°C 30 s, 54–55°C 30 s, and 72°C 45 s, and a final extention for 5 min at 72°C. PCR products were sequenced using an ABI PRISM Dye Terminator Sequencing Kit with AmpliTaq Gold DNA Polymerase (Applied Biosystems Division/Perkin-Elmer, Foster City, CA) and loaded onto an ABI 3100 sequencer. Polymorphism candidates were identified by the PolyPhred program. (P. Green, personal communication) SNP genotypes were verified by manual evaluation of the individual sequence traces.

### Genotyping

Genotyping was completed using SNPstream Ultra High Throughput genotyping system (Beckman Coulter, Fullerton, CA) according to the manufacture's instructions. Briefly, the method combines solution-phase multiplex single nucleotide extension (SNE) with a solid-phase sorting of labeled SNE primers by hybridization to a chip that contains 384 4 × 4 arrays of 12 oligo-nucleotide tags and 4 oligo-nucleotides for positive and negative controls. Each SNE primer contained 1 of the 20 oligonucleotide tags at its 5' end, and the SNE reactions were performed in 12-plex. The microarray plate was imaged by the SNPscope reader (Beckman Coulter, Fullerton, CA). The two-color system allowed the detection of the SNP by comparing signals from the two fluorescent dyes. The image signals were then transferred to genotyping software that translated the images of the arrays into genotype calls. The error rate based upon sequencing for 10% of the SNPs examined in the present study was 0–1.2%.

### Statistical analysis

The Exact Test was calculated to evaluate if the population was in Hardy-Weinberg equilibrium by the Markov chain method, taking *P *< 0.05 as the cutoff for assessing significance. The tagSNPs were chosen on a pairwise basis and linkage disequilibrium (LD) calculation was performed on the confidence interval basis using Haploview 3.2 software.

Genotype and alleles frequencies for each polymorphism were determined by gene counting. Diplotypes of each individual were inferred using the algorithm developed by Stephens et al. (2001) (PHASE). The chi square test was used to determine whether allele frequencies differed between SARS cases and controls. Binary logistic regression was used to analyze the association between a single locus and SARS susceptibility, adjusted for sex and age status, and odds ratio and 95% CI were used to measure strength of association in a genetic risk association study. These statistical analyses were implemented in Statistical Package for the Social Sciences 13.0 software (SPSS Inc, Chicago, Illinois, USA).

## Results

### Demographic characteristics of the population

Demographic characteristics are shown in Table 1. The age and sex distribution of the patients and controls were not significantly different, which indicated that the matching of controls to cases was adequate.

**Table 1:** Demographic characteristics of SARS patients and controls

	SARS patients	Controls	*P*
Beijing population	(*n *= 272)	(*n *= 232)	
Sex			
Male	119(43.8%)	117(50.4%)	0.13
Female	153(56.2%)	115(49.6%)	
Age (years)			
16–30	82(30.1%)	70(30.2%)	0.99
31–40	88(32.4%)	74(31.9%)	
41–50	63(23.2%)	54(23.2%)	
> 50	39(14.3%)	34(14.7%)	
Guangzhou population	(*n *= 104)	(*n *= 291)	
Sex			
Male	46(44.2%)	150(51.5%)	0.20
Female	58(55.8%)	141(48.5%)	
Age (years)			
16–30	33(31.7%)	86(29.6%)	0.73
31–40	35(33.7%)	88(30.2%)	
41–50	21(20.2%)	74(25.4%)	
> 50	15(14.4%)	43(14.8%)	

### Screening *MASP2 *for polymorphisms

**Figure 1:**
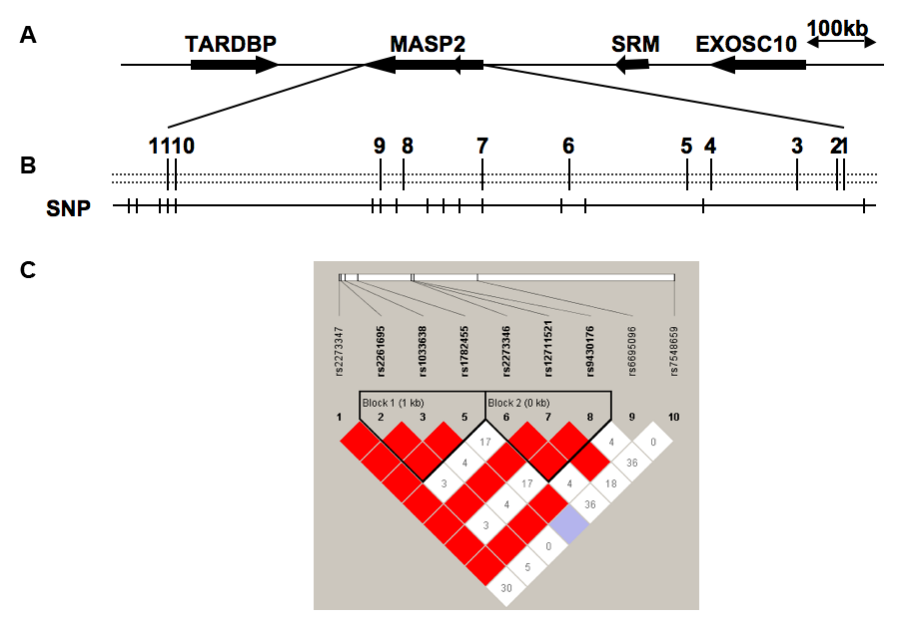
**Gene content of **NC_000001.9**in chromosome 1p36, discovered SNPs and LD of *MASP2***. (a) Genomic structure of genes in this region. (b) Exons of *MASP2 *and the position of SNPs discovered. (c) Pairwise LD between SNPs (MAF >0.05) at this gene. The value within each diamond represents the pairwise correction between SNPs (measured as D') defined by the upper left and upper right sides of the diamond. The diamond without a number corresponds to D' = 1. Shading represents the magnitude and significance of pairwise LD, with a red-to-white gradient reflecting higher to lower LD values.

Sequencing of the 11 exons of *MASP2*, the 5' and 3' regions of the gene, and some intronic sequences in 30 individuals with SARS identified 17 polymorphisms (Table 2). Eleven of the SNPs have been published in the dbSNP database http://www.ncbi.nlm.nih.gov/SNP/index.html. Allele and genotype frequencies were consistent with those expected under Hardy-Weinberg equilibrium. Nine SNPs (allele frequency >5%) were chosen with Haploview for assessment. The SNPs were contained in two blocks of LD (Fig. 1), as defined by Lewontin's| D'|. SNPs including rs7548659, rs2273347 and rs6695096 were located outside of the defined LD blocks. Four tag-SNPs were chosen with the pairwise tagging algorithm implemented in the Tagger program of Haploview: rs7548659, rs12711521, rs2273346 and rs2261695 (r^2 ^threshold was 0.8). Genotype frequencies of the tag-SNP except rs2261695 in other populations that have been published in the HapMap database were shown in table 3 as control. http://www.hapmap.org/cgi-perl/gbrowse/hapmap3_B36/. The four TagSNPs were genotyped in SARS patients and controls with an average success rate of 96%.

**Table 2:** SNPs in MASP2

Region	Nucleotide^a^	dbSNP ID	MAF^b^	Coding polymorphism
5'Up-stream	-153(A/C)	rs7548659	0.230	
intron4	3629(C/T)	-	0.017	
intron5	3934(C/T)	rs3737612	0.017	
intron6	9402(G/T)	-	0.017	
exon7	9420(A/G)	rs12142107	0.017	Ala/Ala
intron7	9736(C/T)	-	0.017	
intron7	9740(G/T)	-	0.017	
intron7	12246(A/G)	rs6695096	0.140	
intron8	16254(C/T)	rs9430176	0.190	
exon9	16369(G/T)	rs12711521	0.350	Tyr/Asp
exon9	16388(C/T)	rs2273346	0.190	Ala/Val
exon11	19594(C/T)	rs1782455	0.130	Ser/Ser
exon11	20009(A/C)	-	0.017	
exon11	20250(A/C)	-	0.017	
3'UTR	20564(C/T)	rs1033638	0.138	
3'Down-stream	20842(G/A)	rs2261695	0.143	
3'Down-stream	20949(T/C)	rs2273347	0.340	

^a^Numbering is from the A of the ATG. ^b^MAF, minor allele frequency

**Table 3:** Genotype frequencies of the three tag-SNP in other populations

SNP^a^	CEU(n = 59)	HCB(n = 45)	JPT(n = 44)	YRI(n = 60)
rs2273346				
Genotype				
TT	100.0%	62.2%	70.5%	70.0%
TC	0.0%	28.9%	25.0%	28.3%
CC	0.0%	8.9%	4.5%	1.7%
rs12711521				
Genotype				
TT	71.7%	37.8%	48.9%	1.6%
TG	28.3%	42.2%	44.4%	16.7%
GG	0.0%	20.0%	6.7%	81.7%
rs7548659				
Genotype				
TT	65.0%	48.9%	65.9%	1.8%
TG	35.0%	42.2%	29.5%	14.0%
GG	0.0%	8.9%	4.5%	84.2%

^a ^The genotype frequencies of rs2261695 were not shown in HapMap database.

### *MASP2 *polymorphisms and susceptibility to SARS-CoV

Genotype data and diplotype (rs2273346 and rs12711521) were analyzed for association with susceptibility to SARS, in Beijing and Guangzhou populations, using Binary logistic regression for overall genotypic association (Tables 4 and 5). No statistically significant evidence of association was observed. The Beijing and Guangzhou sample groups were homogeneous regarding demographic and genetic parameters, so a joined analysis was done, No statistically significant evidence of association was observed (Table 6).

**Table 4:** Genotype and allele frequencies of tagSNPs in MASP2 in SARS patients and controls from Beijing, and their association with risk of SARS

	SARS cases (*n *= 272)	Controls (*n *= 232)	*P *value
rs2261695			
Genotype			
AA	189 (70.3%)	174 (75.0%)	0.49
AG	77 (28.6%)	56 (24.1%)	
GG	3 (1.1%)	2 (0.9%)	
Allele			
A	455 (84.6%)	404 (87.1%)	0.26
G	83 (15.4%)	60 (12.9%)	
rs2273346			
Genotype			
TT	186 (68.4%)	147 (63.4%)	0.19
TC	78 (28.7%)	71 (30.6%)	
CC	8 (2.9%)	14 (6.0%)	
Allele			
T	450 (82.7%)	365 (78.7%)	0.10
C	94 (17.3%)	99 (21.3%)	
rs12711521			
Genotype			
TT	105(38.5%)	106(45.7%)	0.11
TG	137(50.5%)	95(40.9%)	
GG	30(11.0%)	31(13.4%)	
Allele			
T	347 (63.8%)	307(66.2%)	0.43
G	197 (36.2%)	157(33.8%)	
rs7548659			
Genotype			
TT	173 (69.5%)	157 (67.7%)	0.27
TG	61 (24.5%)	67 (28.9%)	
GG	15 (6.0%)	8 (3.4%)	
Allele			
T	407 (81.7%)	381(82.1%)	0.87
G	91 (18.3%)	83(17.9%)	

Diplotype^a^			
TT	344(63.2%)	304(65.5%)	0.29
TG	108(19.9%)	60(12.9%)	
CT	6(1.1%)	2(0.5%)	
CG	86(15.8%)	98(21.1%)	

^a^Two SNP diplotypes span the genomic region of exon 9 (rs2273346 and rs12711521).

**Table 5:** Genotype and allele frequencies of tagSNPs in MASP2 in SARS patients and controls from Guangzhou, northern China, and their association with risk of SARS

	SARS cases (*n *= 104)	Controls (*n *= 291)	*P *value
rs2261695			
Genotype			
AA	73(75.3%)	193(70.4%)	0.45
AG	22(22.7%)	78(28.5%)	
GG	2(2.0%)	3(1.1%)	
Allele			
A	168(86.6%)	464(84.7%)	0.52
G	26(13.4%)	84(15.3%)	
rs2273346			
Genotype			
TT	64(61.5%)	190(66.2%)	0.48
TC	36(34.6%)	82(28.6%)	
CC	4(3.8%)	15(5.2%)	
Allele			
T	164(78.8%)	462(80.5%)	0.61
C	44(21.2%)	112(19.5%)	
rs12711521			
Genotype			
TT	58(55.8%)	139(47.8%)	0.25
TG	36(34.6%)	108(37.1%)	
GG	10(9.6%)	44(15.1%)	
Allele			
T	152(73.1%)	386(66.3%)	0.07
G	56(26.9%)	196(33.7%)	
rs7548659			
Genotype			
TT	70(67.3%)	205(70.5%)	0.15
TG	25(24.0%)	75(27.8%)	
GG	9(8.7%)	11(1.7%)	
Allele			
T	165(79.3%)	485(83.3%)	0.19
G	43(20.7%)	97(16.7%)	

Diplotype^a^			
TT	128(61.5%)	380(65.3%)	0.66
TG	42(20.2%)	76(13.0%)	
CT	4(1.9%)	4(0.7%)	
CG	34(16.4%)	122(21.0%)	

^a^Two SNP diplotypes span the genomic region of exon 9 (rs2273346 and rs12711521).

**Table 6:** The joined analysis of the Beijing and Guangzhou population

	SARS cases		controls				
				
Genotype	P1	P2	C1	C2	P1 vs.P2	C1vs.C2	P1, P2 vs. C1, C2
	(n = 272)	(n = 104)	(n = 232)	(n = 291)	P	P	P
rs2261695							
AA	189 (70.3%)	73 (75.3%)	174 (75.0%)	193 (70.4%)	0.35	0.25	0.76
AG	77 (28.6%)	22 (22.7%)	56 (24.1%)	78 (28.5%)			
GG	3 (1.1%)	2 (2.0%)	2 (0.9%)	3 (1.1%)			
rs2273346							
TT	186 (68.4%)	64 (61.5%)	147 (63.4%)	190 (66.2%)	0.21	0.50	0.63
TC	78 (28.7%)	36 (34.6%)	71 (30.6%)	82 (28.6%)			
CC	8 (2.9%)	4 (3.8%)	14 (6%)	15 (5.2%)			
rs12711521							
TT	105 (38.5%)	58 (55.8%)	106 (45.7%)	139 (47.8%)	0.003	0.64	0.30
TG	137 (50.5%)	36 (34.6%)	95 (40.9%)	108 (37.1%)			
GG	30 (11.0%)	10 (9.6%)	31 (13.4%)	44 (15.1%)			
rs7548659							
TT	173 (69.5%)	70 (67.3%)	157 (67.7%)	205 (70.5%)	0.69	0.50	0.91
TG	61 (24.5%)	25 (24.0%)	67 (28.9%)	75 (27.8%)			
GG	15 (6.0%)	9 (8.7%)	8 (3.4%)	11 (1.7%)			

Diplotype^a^							
TT	344 (63.2%)	128 (61.5%)	304 (65.5%)	380 (65.3%)	0.89	1.00	0.31
TG	108 (19.9%)	42 (20.2%)	60 (12.9%)	76 (13.0%)			
CT	6 (1.1%)	4 (1.9%)	2 (0.5%)	4 (0.7%)			
CG	86 (15.8%)	34 (16.4%)	98 (21.1%)	122 (21.0%)			

P1:SARS patients from Beijing; P2: SARS patients from Guangzhou; C1:controls from Beijing; C2:controls from Guangzhou.

## Discussion

Possible contribution of host genetic factors to the susceptibility and outcome of SARS-CoV infection has been investigated through several association studies [20-23], *MBL *deficiency because of polymorphisms in the *MBL *gene has been shown to be involved in SARS-CoV infection. As the downstream protein of *MBL*, improper MASP activity can interfere with complement functions [24]. there is an association between the genetics of *MASP2 *and its serum level [24-27]. Thiel *et al*. [24] have analyzed the mutation of p.156_159-dupCHNH and SNPs p.R99Q, p.R118C, p.D120G, p.P126L and p.V377A in four populations: Africans from Zambia, Hong Kong Chinese, Brazilian Amerindians and Danish Caucasians. p.156_159-dupCHNH was only found in Chinese (gene frequency 0.26%), associated with low levels of *MASP2*, and p.D120G was found only in Caucasians. p.P126L and p.R99Q were present in Africans and Amerindians only. *MASP2 *levels were reduced in individuals with p.V377A (rs2273346). Therefore, we chose 30 individuals with SARS from Beijing for analysis of *MASP2 *gene polymorphisms. The sample included 60 chromosomes, which provided a 95% confidence level to detect alleles with a frequency >5%. However, we only observed the SNP rs2273346 (p.V377A) among those mentioned in the study of Thiel *et al. *We analyzed four tagSNPs in the *MASP2 *gene in two different SARS patients and controls, searching for a possible genotype-phenotype correction, but no statistically significant difference was found for any polymorphisms between the different groups genotyped, while the possible role of the rare variants is to be determined.

## Conclusion

Our analysis indicates that *MASP2 *polymorphisms is not directly related to SARS-CoV susceptibility in northern and southern Chinese.

## Competing interests

The authors declare that they have no competing interests.

## Authors' contributions

WY have contributed in the preparation of the manuscript and the overall study; YJW and LP performed SNP discovery and data analyses; SYL, YRF and WXY collected and managed DNA samples and clinical information; YX and ZLN have contributed to genotyping; CC, LCX and MQJ performed the data analyses, prepared the manuscript and supervised this study. All authors contribute to writing of the final manuscript. All authors read and approved the final manuscript.

## Pre-publication history

The pre-publication history for this paper can be accessed here:

http://www.biomedcentral.com/1471-2334/9/51/prepub

## Supplementary Material

Additional file 1 Primers used for discovery of *MASP2 *polymorphisms. Click here for file
