# Supplementary material for: Lack of association between polymorphisms of MASP2 and susceptibility to SARS coronavirus infection
Source: BMC Infect Dis. 2009 May 1;9:51. doi: 10.1186/1471-2334-9-51 (PMC2683852; doi:10.1186/1471-2334-9-51)
Supplement: Additional file 1 — Primers used for discovery of MASP2 polymorphisms. [file 1471-2334-9-51-S1.doc]

Additional file 1：Primers used for discovery of *MASP2* polymorphisms

| No | Primer sequence(5'-3') | Annealing temperature(℃) | Amplicon region | Amplicon size(bp) |
| --- | --- | --- | --- | --- |
| 1F | tccttttctgggcacttg | 54 | 5'flanking regions | 704 |
| 1R | cctgagccaaagcgatg |
| 2F | atcacctgtggttgggagt | 54 | 5'flanking regions | 744 |
| 2R | tagaagtcgcttttggctg |
| 3F | tgtggaagaggaagcagc | 55 | 5'flanking regions | 904 |
| 3R | cctgactctgggattctgg |
| 4F | gcagagccaggatttgttt | 54 | 5'flanking regions;exon1 | 557 |
| 4R | ctgacggcaccttgacg |
| 5F | ctggacaaacagatcaaaggtg | 55 | exon1,2; | 457 |
| 5R | tgaaccctggctactccttg |
| 6F | caaggagtagccagggttca | 55 | exon3 | 361 |
| 6R | gcctggcctaagacagagttac |
| 7F | ctgaggtccactctgaggtcat | 55 | exon4 | 471 |
| 7R | accaggtacacagtgggatgtta |
| 8F | cacatccctgtcccagtttc | 54 | exon5 | 481 |
| 8R | ctctgcctcccacacttgtact |
| 9F | agtacaagtgtgggaggcagag | 55 | exon6 | 393 |
| 9R | agccctacactctacagctcctt |
| 10F | cttcagatggctcgattcag | 54 | exon7 | 480 |
| 10R | caccagctttccaaactagc |
| 11F | gccaatacctgttgagtgatga | 55 | exon8 | 391 |
| 11R | acagtagcagcagagggagttc |
| 12F | catttagttacctcccacctcag | 54 | exon9 | 423 |
| 12R | ggctcaagttccaagtattgc |
| 13F | ttcagacacctctatgtcccact | 55 | exon10 | 279 |
| 13R | cacagctaaagctctcctcactg |
| 14F | aggtagtcacccaccaattc | 54 | exon11 | 570 |
| 14R | ctttgggttaatccccatcc |
| 15F | tcatgatgctggctttgac | 55 | exon11 | 473 |
| 15R | tgttctcgatccagggaata |
| 16F | gtgaaacagagaggtggtttgtg | 55 | exon11; | 470 |
| 16R | catggacaggcagtttacagaa |
| 17F | ccagccttacccattgactc | 55 | exon11;  3’ flanking regions | 484 |
| 17R | tgccaccctccctgaacat |
